# Flexible bio-memristive devices based on chicken egg albumen:Au@SiO_2_ core-shell nanoparticle nanocomposites

**DOI:** 10.1038/s41598-017-12209-6

**Published:** 2017-09-20

**Authors:** Chang Han Bok, Sung Jun Woo, Chaoxing Wu, Jae Hyeon Park, Tae Whan Kim

**Affiliations:** Department of Electronics and Computer Engineering, Hanyang University, Seoul, 04763 Republic of Korea

## Abstract

Flexible bio-memristive (FBM) devices utilizing chicken egg albumen (CEA):Au@SiO_2_ core-shell nanoparticle nanocomposites were fabricated on indium-tin-oxide (ITO) coated polyethylene naphthalate (PEN) substrates. Current-voltage (I-V) curves for the Al/CEA:Au@SiO_2_ core-shell nanoparticle/ITO/PEN devices showed clockwise current hysteresis behaviors due to the existence of the CEA:Au@SiO_2_ core-shell nanoparticle nanocomposites. The endurance number of the ON/OFF switching for the FBM devices was above 10^2^ cycles. An ON/OFF current ratio of 1 × 10^5^ was maintained for retention times longer than 1 × 10^4^ s. The memory characteristics of the FBM devices after bending were similar to those before bending. The memory margin and the stability of FBM devices were enhanced due to the embedded Au@SiO_2_ core-shell nanoparticles. The switching mechanisms occurring in the Al/CEA:Au@SiO_2_ core-shell nanoparticle/ITO-coated PEN devices are described on the basis of the I-V results and the filament mechanisms.

## Introduction

Organic/inorganic nanocomposites have been extensively used for potential applications in light-emitting devices, nonvolatile memory (NVM) devices, solar cells, and nanogenerators^1–4^. The electrical characteristics and the memory mechanisms of NVM devices fabricated with organic/inorganic hybrid nanocomposites have been particularly interesting due to those devices having superior advantages of good mechanical flexibility, low cost, low power consumption, and simple fabrication^5,6^. Among the several kinds of organic materials, chicken egg albumen (CEA) has recently emerged as a novel candidate due to its promising applications in next-generation NVM devices and its human-friendly properties. CEA consists of water (88.5%), protein (0.5%), carbohydrate (0.5%), and other minerals (0.5%)0^7^. The protein of CEA is composed of ovalbumin (54%), conalbumin (12%), ovomucoide (11%), and lysozyme (3.5%)^8^. These proteins become denatured when a large amount of heat energy is applied. The denaturation of the proteins changes the paths of oxygen diffusion and reduces the probability of oxygen scattering, resulting in an increase in the possibility of forming and rupturing conductive filaments in NVM devices^9^. Because the dielectric constant of CEA is larger than those of typical organic materials, such as poly(methyl methacrylate) (PMMA) and polystyrene (PS), the current drivability of the CEA-based transistor is higher than those of the typical organic-material-based transistors^10^. Even though the surface smoothness of CEA is similar to those of PMMA and PS, CEA is cheaper, and memristive devices using CEA are simpler to fabricate than those using PMMA and PS because CEA can be easily obtained from eggs without any synthesis or extraction^11^. Furthermore, because CEA is biodegradable, bioresorbable, eco-friendly, and mechanically flexible, it has been considered to be one of the most suitable materials for electronic systems attached to wearable and bio-devices. For these reasons, CEA has been extensively used as a dielectric or active material in thin-film transistors, synaptic devices, and memristive devices^10–12^. In addition, resistive-switching bio-memory devices based on the protein of silk fibroin and the chitosan of crab shells have been extensively studied^13–15^.

Because Au nanoparticles (NPs) have excellent electrical, optical, and catalytic properties among the various kinds of inorganic materials, they have been used as conductive materials^16–19^. When the annealing temperature of the active layer of the NVM devices is 120 °C, Au NPs might be clumped together. As a solution to this problem, silica-coated Au (Au@SiO_2_) core-shell NPs, instead of Au NPs, have been used as an active layer with the albumen. Because the shell part of the Au NPs is made of SiO_2_, the heat resistance of the Au@SiO_2_ is higher than that of typical Au NPs^20^. Even though some works concerning the electrical characteristics of NVM devices utilizing CEA organic materials have been performed^9,21^, studies on the electrical stabilities of and the carrier transport mechanisms in flexible bio-memristive (FBM) devices fabricated utilizing CEA/Au@SiO_2_ core-shell nanocomposites have not been conducted yet.

This paper presents data for the electrical stabilities of and the carrier transport mechanisms in eco-friendly, flexible memory devices based on CEA:Au@SiO_2_ core-shell nanocomposites. Scanning electron microscopy (SEM) measurements were performed to analyze the structural properties of the CEA/Au@SiO_2_ core-shell nanocomposites. Transmission electron microscopy (TEM) images and energy dispersive X-ray spectroscopy (EDS) mapping images were measured to confirm the chemical elements in the CEA:Au@SiO_2_ active layer. Current-voltage (I-V) measurements were performed before and after bending to investigate the electrical bistabilities of the FBM devices. The number of endurance cycles and the retention times for the devices were measured before and after bending as a demonstration of the stability of the devices. The carrier transport mechanisms in the FBM devices fabricated with CEA:Au@SiO_2_ core-shell NP nanocomposites are described on the basis of the I-V curves and filament mechanisms.

## Methods

**Figure 1:**
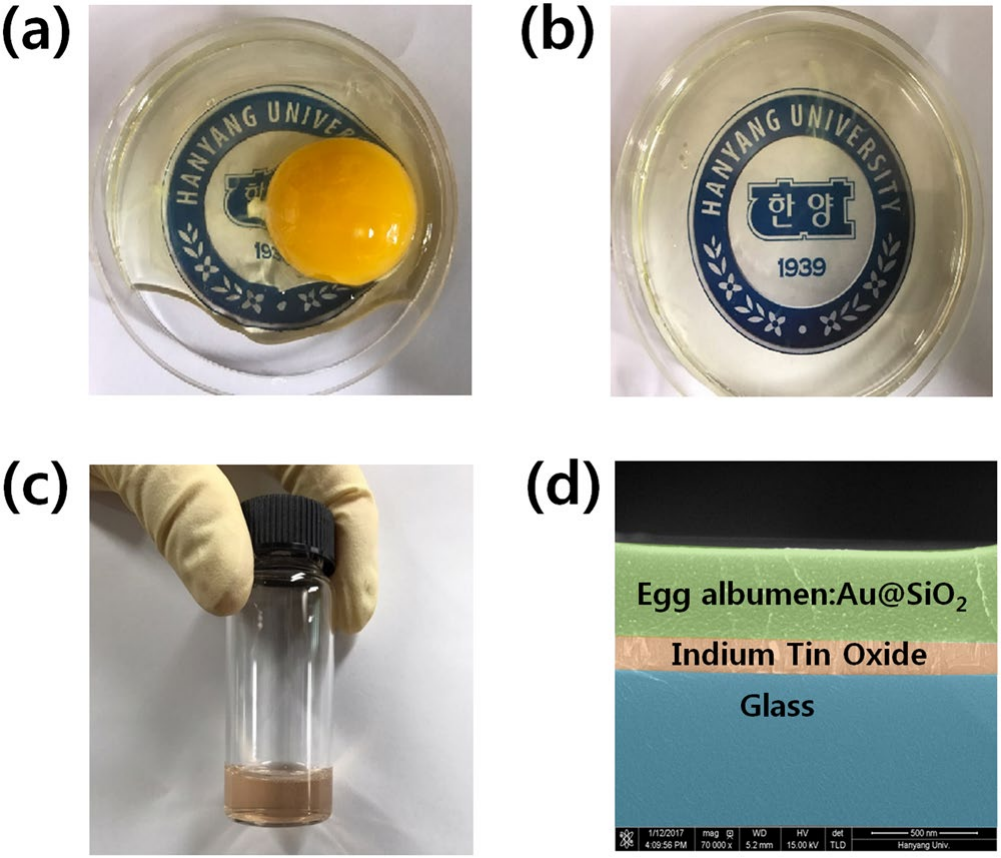
Photographs of the (**a**) entire egg consisting of an egg white and an egg yolk, the (**b**) egg albumen liquid, and the (**c**) egg albumen:Au@SiO_2_ solution (**d**) cross-sectional scanning electron microscopy image of the egg albumin: Au@SiO_2_ on an ITO-coated glass substrate.

Figure 1 shows the solution fabrication process for the FBM devices used in this work. Figure 1(a) shows an entire egg consisting of egg white and egg yolk. The CEA liquid was separated by using a steel mesh spoon. Figure 1(b) shows separated CEA liquid. The separated CEA liquid was mixed with a Au@SiO_2_ core-shell solution (purchased from Aldrich Co.) in a volume ratio of 1:9. Then, ultrasonic processing was performed on the CEA:Au@SiO_2_ solution for 15 min at room temperature. The Au NPs had diameters of 5 nm. The Au NPs were dispersed in a H_2_O solution, and the concentration of the Au NPs was approximately 6.6 × 10^13^ particles/mL.

Indium-tin-oxide (ITO)-coated glass substrates were alternately cleaned by using a chemical cleaning procedure, with methanol, and deionized water for 20 min each. ITO-coated polyethylene naphthalate (PEN) substrates were cleaned with methanol and deionized water for 20 min each in that order. Cleaned substrates were dried by using N_2_ gas with a purity of 99.999%. Then, the ITO-coated glass and PEN substrates were treated in an ultraviolet-ozone cleaner for 20 min. A prepared CEA:Au@SiO_2_ solution was spin coated onto the cleaned ITO-coated glass and PEN substrates at 4000 rpm. Figure 1(d) shows a cross-sectional SEM image of the CEA:Au@SiO_2_ core-shell nanocomposite formed on an ITO-coated glass substrate. The thickness of the CEA:Au@SiO_2_ core-shell nanocomposite film was about 377 nm. After spin coating, the devices were annealed on a hotplate at 120 °C for 20 min because the protein becomes denatured during a high-temperature treatment, result in an improved switching performance^9^. Finally, the Al top electrode was deposited to a thickness of 150 nm on the active layer by using thermal evaporation at a system pressure of 1 × 10^6^ Torr.

## Results and Discussion

**Figure 2:**
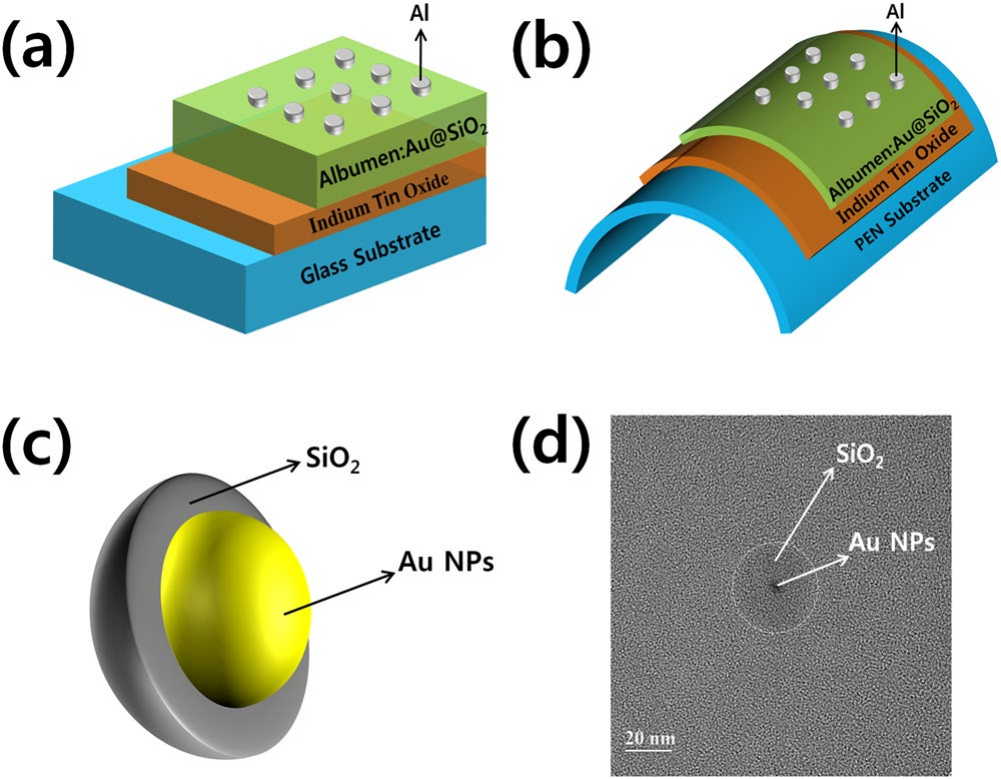
Schematic diagrams of the fabricated devices with Al/egg albumen:Au@SiO_2_/ITO-coated (**a**) glass and (**b**) PEN substrates. (**c**) Schematic diagram of the Au@SiO_2_ core-shell, and (**d**) transmission electron microscopy image of the Au@SiO_2_ core-shell.

Figure 2(a) and (b) show schematic diagrams of the Al/CEA:Au@SiO_2_/ITO/glass device and the flexible Al/CEA:Au@SiO_2_/ITO/PEN device, respectively. The structure of the Au@SiO_2_ core-shell is shown in Fig. 2(c). Au NPs are surrounded by SiO_2_ shells, which protect the Au NPs from external energy such as heat. Figure 2(d) shows a TEM image of the Au@SiO_2_ core-shell, which demonstrates that SiO_2_ shells are wrapped around the Au NPs.

**Figure 3:**
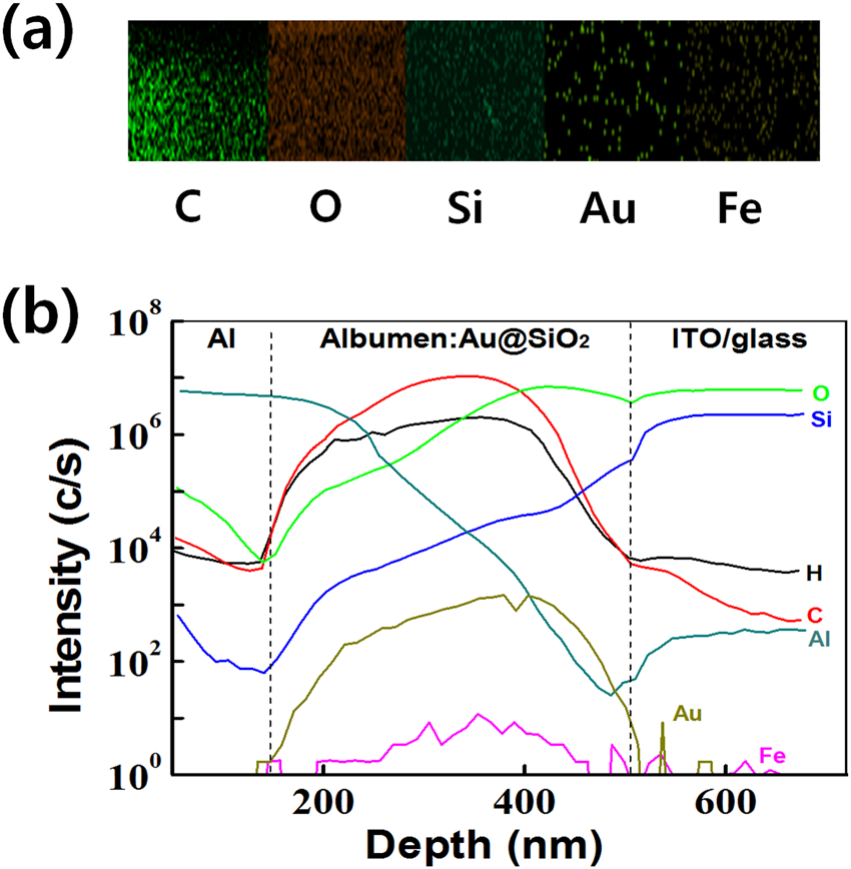
(**a**) Energy dispersive X-ray spectroscopic mapping image and (**b**) time-of-flight secondary ions mass spectroscopy depth profiling of the albumen:Au@SiO_2_ layer.

Figure 3(a) shows EDS mapping images of the CEA:Au@SiO_2_ layer. The EDS data are obtained in the cross-sectional mode of the transmission electron microscope. The EDS data show the distributions of the chemical elements in the CEA:Au@SiO_2_ active layer. Elemental C, O, Si, Au, and Fe were observed in the CEA:Au@SiO_2_ layer. Figure 3(b) shows the results of time-of-flight secondary ion mass spectrometry (TOF-SIMS). The TOF-SIMS data for the Al/CEA:Au@SiO_2_/ITO/glass memory devices showed that the Au@SiO_2_ had penetrated into the CEA. Each element obtained through the component analysis plays an important role in the resistive-switching performance of the Al/CEA:Au@SiO_2_/ITO devices.

**Figure 4:**
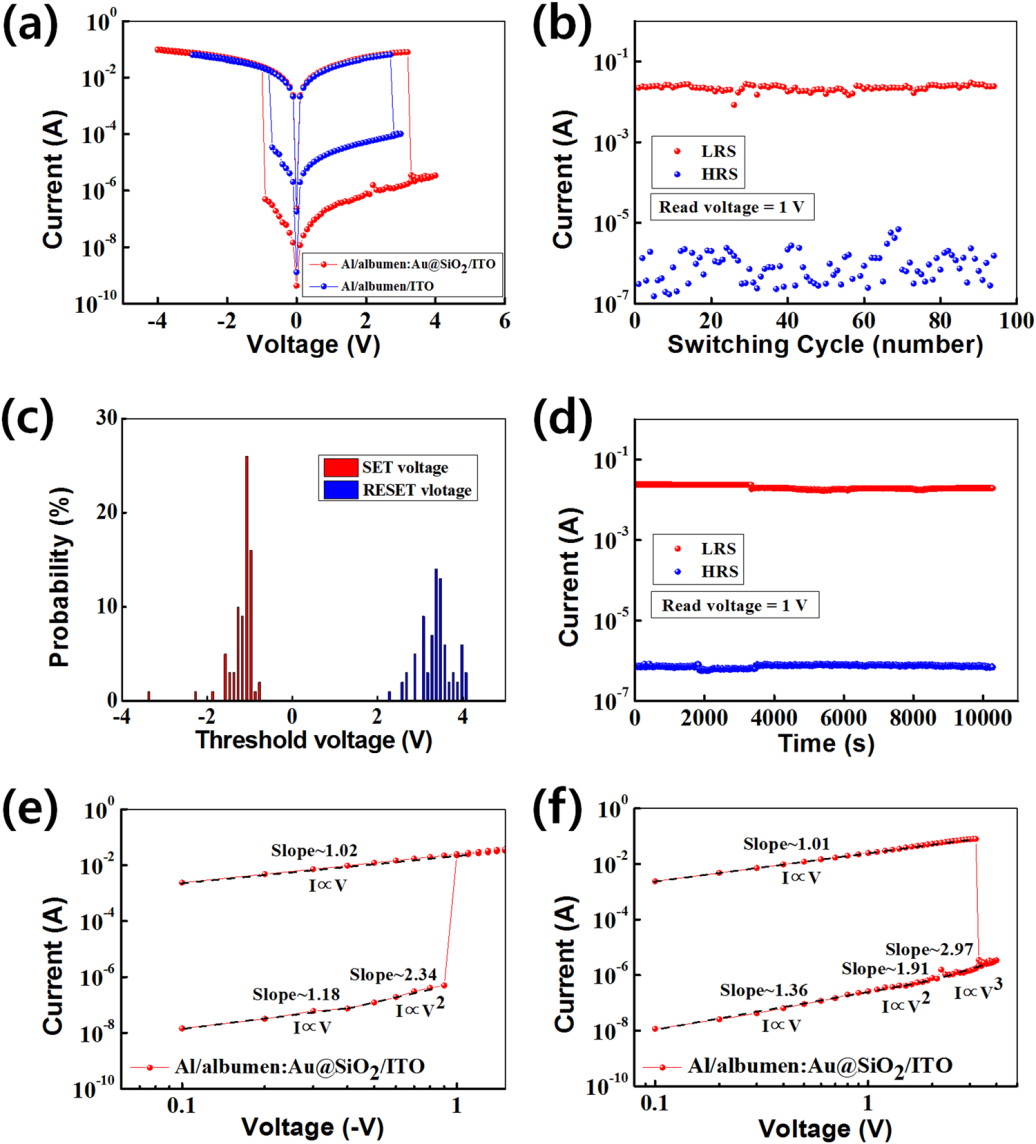
(**a**) Current-voltage curves of the Al/egg albumen:Au@SiO_2_/ITO/glass devices. (**b**) Number of endurance cycles, (**c**) probability distributions of the SET and the RESET threshold voltages, (**d**) retention times, (**e**) data fitting the ON state, and (**f**) data fitting the OFF state for the devices.

Figure 4(a) shows the I-V curves of the fabricated bio-memristive devices. The blue and the red lines represent I-V curves for the Al/CEA/ITO and the Al/CEA:Au@SiO_2_/ITO devices, respectively. The I-V curves exhibit the hysteresis characteristics of bio-memristive devices. When sweeping voltages were applied to the Al/CEA:Au@SiO_2_/ITO devices, the conducting states were identified as at a low-resistance (ON) state and a high-resistance (OFF) state. The OFF state of the bio-memristive devices was maintained at applied voltages between 0 and −0.9 V. When an applied voltage of −1.0 V (V_SET_) was applied, the current of the memory device rapidly increased from 4.98 × 10^−7^ to 2.25 × 10^−2^ A, which was associated with the SET process. The ON state of the memory device was maintained for applied voltages from −1.1 to −4 V and from −4 to 3.2 V. When an applied voltage of 3.3 V (V_RESET_) was applied, the current of the bio-memristive device rapidly decreased from 7.92 × 10^−2^ to 3.48 × 10^−6^ A, which was equivalent to the RESET process. The OFF state of the device was maintained for applied voltages from 3.4 to 4 V and from 4 to 0 V. The ON/OFF current ratio of the Al/CEA:Au@SiO_2_/ITO devices was larger than that of the Al/CEA/ITO devices. The current in the Al/CEA/ITO device rapidly increased from 3.35 × 10^−5^ to 1.83 × 10^−2^ A when a voltage of −0.8 V was applied to the device. When a voltage of 2.8 V was applied to the memory device, the current rapidly decreased from 6.45 × 10^−2^ to 9.92 × 10^−5^ A. The Au NPs embedded in the CEA matrix reduced the electric field at the electrode and obstructed charge injection in the OFF state of the bio-memristive device, resulting in the occurrence of space charge effects^22,23^. Therefore, the ON/OFF current ratio of the Al/CEA:Au@SiO_2_/ITO device was two orders of magnitude larger than that of the Al/CEA/ITO memory device.

Figure 4(b) shows the currents as functions of the number of cycles for the ON and the OFF states of the Al/CEA:Au@SiO_2_/ITO devices. The endurance data showed the existence of resistance states while applying a voltage to a memory device in the ON and the OFF states until 100 cycles at a read voltage of 1 V. The ON state maintained a current of 2 × 10^2^ A up to at least 100 cycles without any significant current change. The OFF state data showed the fluctuation in the current between 7.29 × 10^−7^ and 9.84 × 10^−6^ A for up to 100 sweeping cycles. The scatter in the current in the OFF state originates from the inhomogeneity and the random rupture of conductive filaments^21^. Whenever conductive filaments are destroyed, the positions and the degree of the conductive filaments are randomly distributed. After the RESET process has been achieved, a difference in the values of the OFF current is observed^21,24^. However, the Al/CEA:Au@SiO_2_/ITO devices can be utilized as resistive-memory devices because the ON/OFF current ratio is sufficiently high^25,26^. Fig. 4(c) shows the probability distributions of the threshold voltages for the set voltage (V_SET_) and for the reset voltage (V_RESET_) for the Al/CEA:Au@SiO_2_/ITO devices. The V_SET_ threshold voltages are distributed between −2 and −1 V, and the distribution of the V_SET_ threshold voltages is localized around −1.1 V. The V_RESET_ threshold voltages are widely distributed between 3 and 4 V.

Figure 4(d) shows the retention characteristics of the Al/CEA:Au@SiO_2_/ITO devices. A read voltage of 1 V was applied to the memory device to determine the retention time of the device. The retention data were measured over 1 × 10^4^ s to confirm the stability of the memory device. The retention results demonstrate excellent device stability. Figure 4(e) and (f) show the I-V fitting results for the ON and the OFF states, respectively, which were performed to understand the current-transport mechanisms in the device. The I-V curves for the ON and the OFF states were replotted on a double nature logarithmic scale. The slope of the I-V fitting curves for the OFF state was approximately 1 at low voltages for the ON and the OFF states, indicating that the current-transport mechanism was dominated by Ohm’s law. The slopes of the I-V curves for ON state at high voltages above 0.4 V and for the OFF state at medium voltages between 1.5 and 2.1 V were approximately 2, indicating that the current-transport mechanisms were related to the Mott-Gurney law^27^ and Child’s law^28,29^. The slope of the OFF state at high voltages above 2.2 V was approximately 3, indicating that the current-transport mechanism was dominated by the trap-controlled space-charge-limited current (TC-SCLC) and the trap-filling process^27,30^.

**Figure 5:**
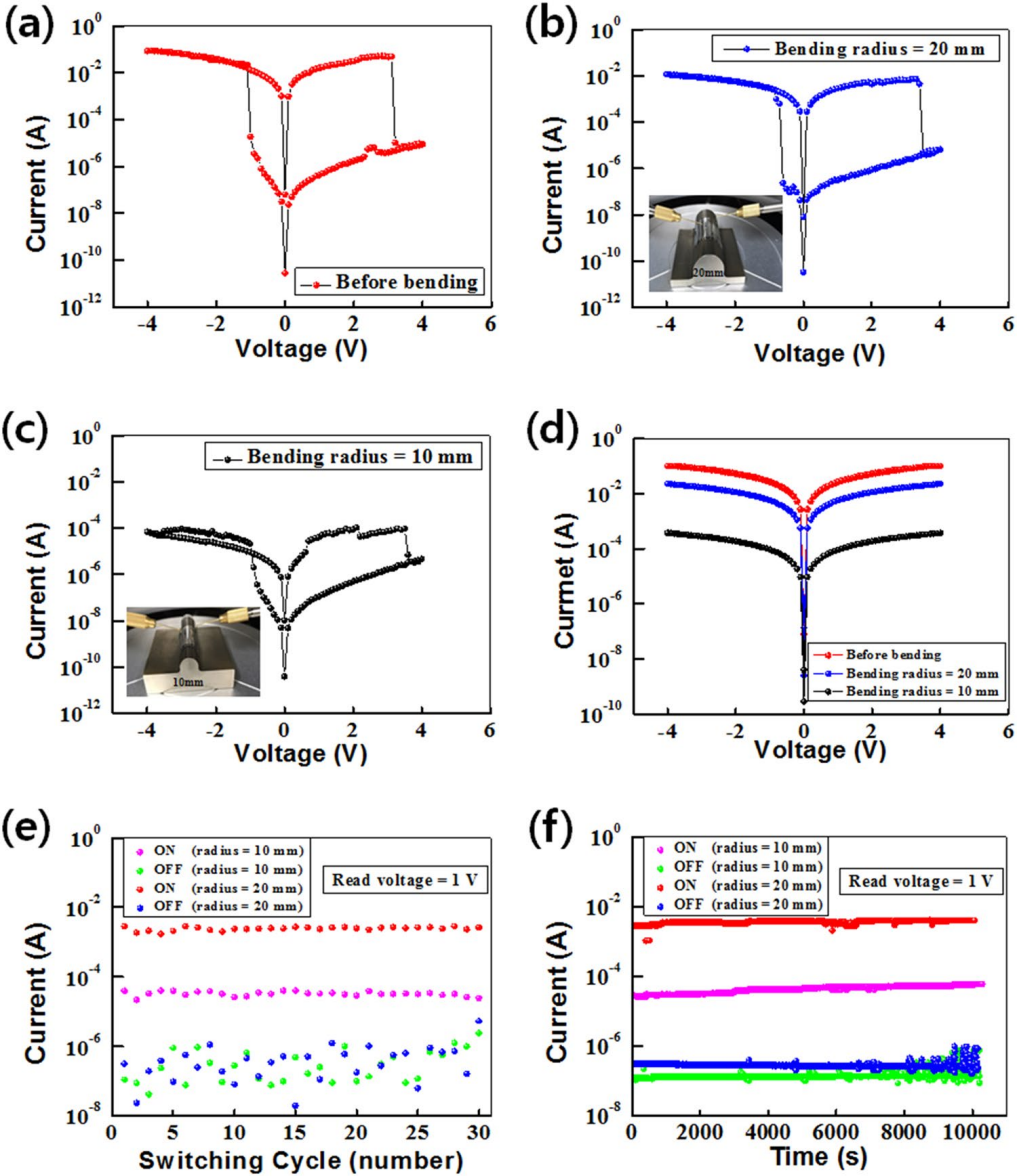
Current-voltage curves of the Al/egg albumen:Au@SiO_2_/ITO/PEN devices (**a**) before bending and after bending with radii of (**b**) 20 and (**c**) 10 mm. (**d**) ON current according to the radius at the contact on the ITO layer, (**e**) endurance cycling, and (**f**) retention time after bending for the devices.

Figure 5 shows the I-V characteristics of the FBM devices fabricated on ITO-coated PEN substrates. The I-V curves of the FBM devices demonstrated three states with bistable resistive-switching behaviors. The I-V characteristics of the Al/CEA:Au@SiO_2_/ITO/PEN device before bending were similar to those of the Al/CEA:Au@SiO_2_/ITO/glass device, as shown in Fig. 5(a). Even though the I-V curves of the memory device at a bending radius of 20 mm showed no electrical noise, the “ON” current decreased, as shown in Fig. 5(b). The ON state current for the device measured at a bending radius of 10 mm was smaller than that of the device measured at a bending radius of 20 mm, as shown in Fig. 5(c). The I-V characteristics of the memory devices with a bending radius of 10 mm showed slight electrical noise and a low ON/OFF ratio, which originated from the decrease in the resistance of the ITO bottom electrode due to the bent PEN substrate. The decrease in the ON state current after bending is due to an increase in the resistance of the ITO layer, as shown in Fig. 5(d). That decrease in the ON state current might have been caused by defects in the ITO originating from the mechanical bending of the devices. Figure 5(e) shows endurance-test data for the Al/CEA:Au@SiO_2_/ITO/PEN devices after bending. The magenta and the green dots represent the ON and the OFF states, respectively, of the devices for a bending radius of 10 mm. The red and the blue dots represent the ON and the OFF states, respectively, of the devices for a bending radius of 20 mm. The electrical characteristics of the devices fabricated utilizing CEA:Au@SiO_2_ core-shell nanocomposites showed superior endurance characteristics under bending.

Figure 5(f) shows the retention properties of the Al/CEA:Au@SiO_2_/ITO/PEN devices under bending. The magenta and the green lines represent the ON and the OFF states, respectively, of the devices for a bending radius of 10 mm. The red and the blue lines represent the ON and the OFF states, respectively, for a bending radius of 20 mm. The retention characteristics of the devices fabricated utilizing CEA:Au@SiO_2_ core-shell nanocomposites under bending were stably maintained without any significant degradation for up to 1 × 10^4^ s. Even in the bent state, the electrical characteristics of the devices exhibited excellent stabilities, as shown in Fig. 5(e) and (f).

The electrical characteristics of a variety of bio-compatible memristive devices are summarized in Table 1. The data in that table show that, compared to other bio-compatible memristive devices, the FBM devices fabricated in this work have very reliable switching properties. A further comparison to those devices based on other biomaterials shows that our FBM devices fabricated utilizing CEA:Au@SiO_2_ nanocomposites have outstanding performance at lower cost; moreover, the process for their fabrication is simpler.

**Table 1:** Electrical characteristics of bio-memristive devices fabricated utilizing various biomaterials.

Active layer	ON/OFF ratio	Retention (s)	Endurance (cycle)	References
CEA:Au@SiO_2_	>10^5^	>10^4^	>10^2^	This work
Silk Fibroin Protein	~10	>800	>1200	13,14
Silk Protein:Au Nanoparticles	>10^6^	>10^2^	>10	37
Enzyme Multilayers	>10^2^	>10^4^	>200	38
PAH:Ferritin Nanoparticle Multi-Layers	>10^3^	>10^4^	>300	35
Sericin	~10^6^	>10^4^	~21	24
Nanocellulose	>10^7^	~10^4^		39

**Figure 6:**
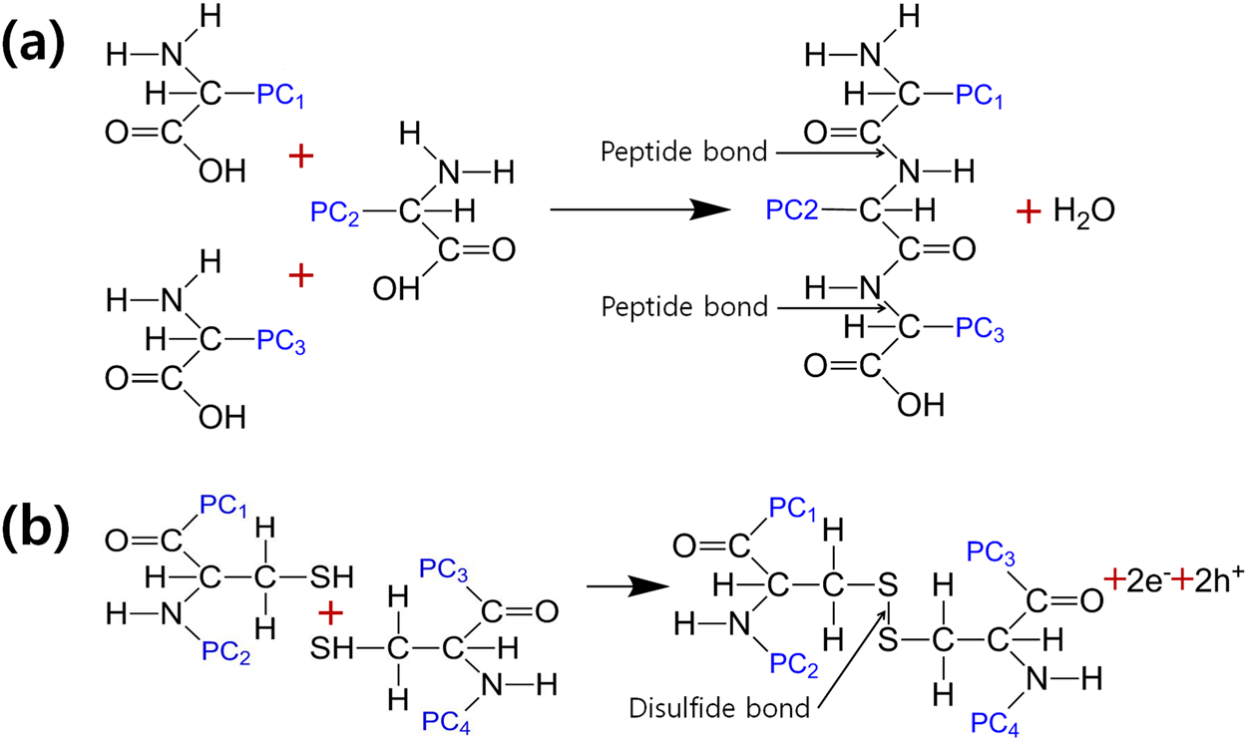
Scheme for the formation processes of the (**a**) peptide bonds and the (**b**) disulfide bonds between amino acids on different protein chains.

The proteins of CEA are comprised of amino acids with long protein chains, and those proteins in CEA are connected together by weak chemical bonds. While the weak bonds are disconnected during the thermal process, protein molecules are cross-linked with two types of major chemical bonds, peptide bonds and disulfide bonds. Figure 6(a) and (b) show the formation processes of those two major bonds. The denaturation of proteins can be irreversible, called coagulation^11,31^, and the most important chemical reaction of irreversible denaturation involves the disulfide bonds forming so-called disulfide bridges between two cross-linked protein molecules. The leakage current in a thermally self-crosslinked CEA film is reduced by the formation of these bridges^11^. Water molecules, electrons, and hydrogen ions are generated during this process, as well.

**Figure 7:**
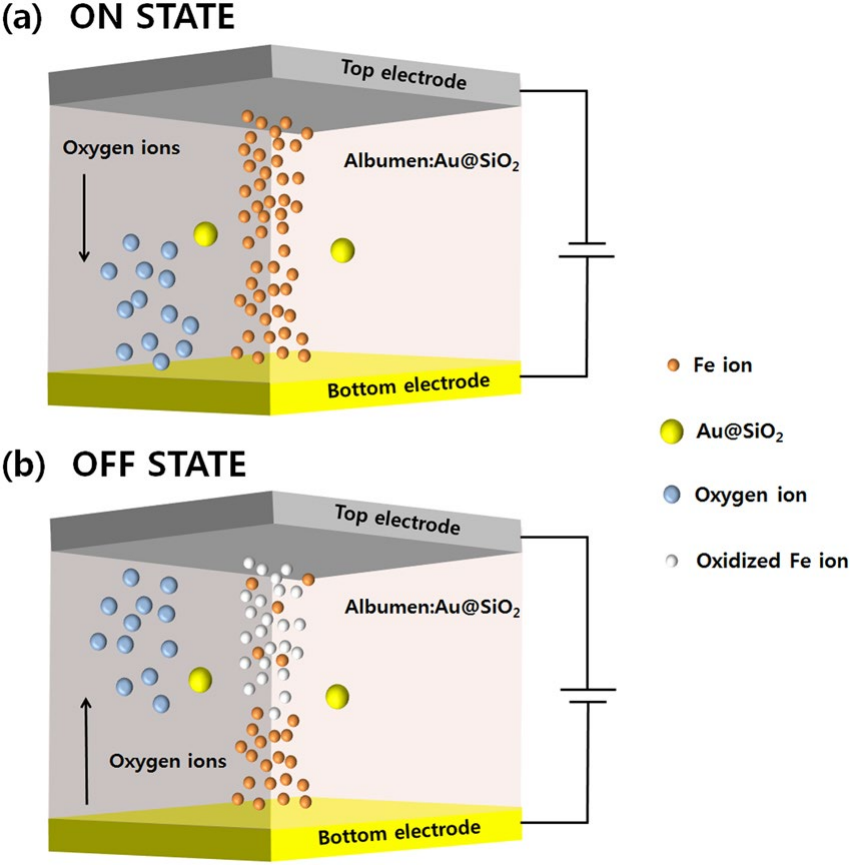
Filament mechanisms of the (**a**) ON and the (**b**) OFF states for the devices.

Electrons cannot transfer easily without a mediator between the redox-active centers of the protein due to the electrochemical property of protein-based materials. For this reason, electrochemical modifications involving the surfaces of the electrodes and the transition-metal ions must take place because of the redox reaction^32^. Fe ions are suitable for electron transfer between the top and the bottom electrodes due to the small difference in the work functions between the Al and the ITO electrodes. Among the approximately 40 different proteins, conalbumin is ovotransferrin, which is an iron-binding protein with high sensitivity to heat^9,33,34^. Fig. 7(a) and (b) show the switching mechanisms of the devices containing a CEA/Au@SiO_2_ active layer. When negative voltages are applied to the top electrode, electrons are injected into the CEA due to the low work function and the injected electrons fill trapping defects in the insulator^21^. Additionally, the amino acids from decomposition of the proteins in the CEA/Au@SiO_2_ active layers can absorb metal ions, resulting in the formation of conductive filaments^35,36^. The oxygen ions diffuse from the top electrode to the bottom electrode with increasing negative applied voltage, and the number of oxidized Fe ions in the active layer decreases. Because conductive filaments are formed between the top and the bottom electrodes, the electrons easily move along the filaments, resulting in a rapid increase in the current, which is equivalent to the SET process for the Al/CEA:Au@SiO_2_/ITO devices. When positive voltages are applied to the devices for a RESET process, the oxygen ions diffuse from the bottom electrode to the top electrode. Then, Fe ions are oxidized by diffusing oxygen ions, and the filaments are ruptured. When the filaments are ruptured, the resistance state of the devices changes from the ON to the OFF state^9^.

## Conclusion

Eco-friendly, FBM devices were fabricated utilizing CEA:Au@SiO_2_ nanocomposites. The ON/OFF current ratio of the devices fabricated utilizing the CEA:Au@SiO_2_ nanocomposites was larger than that of the devices based only on albumen. The memory characteristics of the FBM devices after bending were almost the same as those of the devices before bending. The retention results and the endurance data for the Al/CEA:Au@SiO_2_/ITO devices demonstrated stable and reproducible operation. These results indicate that FBM devices based on CEA:Au@SiO_2_ hold promise for applications in next-generation, eco-friendly, FBM devices.
